# To Vaccinate or Not to Vaccinate—opinion

**DOI:** 10.1155/S1064744995000196

**Published:** 1995

**Authors:** William J. Ledger


					Infectious Diseases in Obstetrics and Gynecology 2:282 (1995)
(C) 1995 Wiley-Liss, Inc.
To Vaccinate or Not to
Vaccinate---Opinion
I agree with Dr. Faro's concern about hepatitis B virus infection and his emphasis
upon the hepatitis B vaccine. I would like to emphasize universal coverage, beyond
the focus upon sexually active adolescents. This population should be cared for
because of their high-risk sexual activity. However, they should not be our sole
concern. Approximately half of our married population in their 20s and 30s get
divorced. I've been impressed that divorce for many women is followed by a
period of sexual activity with more than one sexual partner. Obstetrician-gynecol-
ogists should be proponents for universal hepatitis B immunization for all women.
As advocates for the best health care for women, we should not be satisfied with
anything less.
Sincerely,
William J. Ledger, M.D.
Letter to the Editor
Received February 5, 1995
Accepted February 17, 1995

				

